# Screening and brief intervention training for HIV health-care workers in sub-Saharan Africa

**DOI:** 10.1186/1940-0640-7-S1-A72

**Published:** 2012-10-09

**Authors:** Richard Spence

**Affiliations:** 1 School of Social Work, University of Texas, Austin, TX, USA

## 

Screening and feedback, brief intervention, and motivational interviewing have shown efficacy for reducing alcohol consumption. However, current protocols have been designed for Western settings. The present study adapted the methods to cultural and socioeconomic conditions in three developing African countries (Botswana, Tanzania, and Namibia). An intervention and associated curriculum was developed to train health-care workers to screen all patients and deliver a brief motivational intervention (BMI). A pocket guide and patient handouts were developed as implementation aids. Local beverages were referenced as standardized equivalents of beer, wine, and liquor. Materials were produced in English and prevalent local languages. Ministries of Health in each country adopted the methods as a strategy to reduce risky alcohol consumption and associated HIV infection risk in targeted areas. Based on follow-up surveys 60 days after the training, 64% of health-care workers in Tanzania and 94% in Namibia began using BMI for an average of 25% and 30% of their patients. Based on 14 clinician respondents in each country, a total of 252 Tanzanian patients and 424 Namibian patients received BMI in the two months following training. Implementation barriers included time, perceived stigma, and perceived patient resistance. The training and follow-up surveys document the health-care workers’ need for a culturally relevant brief intervention to address risky alcohol use in an HIV prevention setting in sub-Saharan Africa. Follow-up plans include streamlined delivery methods, implementing monitoring and evaluation tools, and providing refresher training sessions.

